# HIV-Infected Individuals with Low CD4/CD8 Ratio despite Effective Antiretroviral Therapy Exhibit Altered T Cell Subsets, Heightened CD8+ T Cell Activation, and Increased Risk of Non-AIDS Morbidity and Mortality

**DOI:** 10.1371/journal.ppat.1004078

**Published:** 2014-05-15

**Authors:** Sergio Serrano-Villar, Talia Sainz, Sulggi A. Lee, Peter W. Hunt, Elizabeth Sinclair, Barbara L. Shacklett, April L. Ferre, Timothy L. Hayes, Ma Somsouk, Priscilla Y. Hsue, Mark L. Van Natta, Curtis L. Meinert, Michael M. Lederman, Hiroyu Hatano, Vivek Jain, Yong Huang, Frederick M. Hecht, Jeffrey N. Martin, Joseph M. McCune, Santiago Moreno, Steven G. Deeks

**Affiliations:** 1 Department of Infectious Diseases, University Hospital Ramón y Cajal, Madrid, Spain; 2 Molecular Immune Biology Laboratory, University Hospital Gregorio Marañón, Madrid, Spain; 3 Department of Medicine, University of California San Francisco, San Francisco, California, United States of America; 4 Department of Medical Microbiology and Immunology, School of Medicine, University of California Davis, Davis, California, United States of America; 5 Department of Epidemiology, Johns Hopkins University, Baltimore, Maryland, United States of America; 6 Case Western Reserve University, Cleveland, Ohio, United States of America; 7 Bioengineering and Therapeutic Sciences, University of California San Francisco, San Francisco, California, United States of America; 8 Department of Epidemiology and Biostatistics, University of California San Francisco, San Francisco, California, United States of America; Emory University, United States of America

## Abstract

A low CD4/CD8 ratio in elderly HIV-uninfected adults is associated with increased morbidity and mortality. A subset of HIV-infected adults receiving effective antiretroviral therapy (ART) fails to normalize this ratio, even after they achieve normal CD4+ T cell counts. The immunologic and clinical characteristics of this clinical phenotype remain undefined. Using data from four distinct clinical cohorts and three clinical trials, we show that a low CD4/CD8 ratio in HIV-infected adults during otherwise effective ART (after CD4 count recovery above 500 cells/mm^3^) is associated with a number of immunological abnormalities, including a skewed T cell phenotype from naïve toward terminally differentiated CD8+ T cells, higher levels of CD8+ T cell activation (HLADR+CD38+) and senescence (CD28− and CD57+CD28−), and higher kynurenine/tryptophan ratio. Changes in the peripheral CD4/CD8 ratio are also reflective of changes in gut mucosa, but not in lymph nodes. In a longitudinal study, individuals who initiated ART within six months of infection had greater CD4/CD8 ratio increase compared to later initiators (>2 years). After controlling for age, gender, ART duration, nadir and CD4 count, the CD4/CD8 ratio predicted increased risk of morbidity and mortality. Hence, a persistently low CD4/CD8 ratio during otherwise effective ART is associated with increased innate and adaptive immune activation, an immunosenescent phenotype, and higher risk of morbidity/mortality. This ratio may prove useful in monitoring response to ART and could identify a unique subset of individuals needed of novel therapeutic interventions.

## Author Summary

The CD4/CD8 ratio, a hallmark of the collection of T cell defects related to aging –“immunosenescence”- and a predictor of mortality in the general population, often fails to normalize in an important proportion of HIV-infected individuals with adequate CD4+ T cell recovery after ART initiation. However, the immunological and clinical characteristics of this clinical phenotype have not been elucidated. Herein we show that during treated HIV infection, expansion of CD8+ T cells, reflected as a low CD4/CD8 ratio, identifies a subgroup of individuals with a number of immunological abnormalities and a poor prognosis. These subjects exhibit increased innate and adaptive immune activation, an immunosenescent phenotype, CD4+ and CD8+ imbalance in the gut mucosa and higher risk of morbidity and mortality. In contrast, those who normalize the CD4/CD8 ratio have traits of a healthy immune system. We observed that early ART initiation might contribute to more rapid and robust CD4/CD8 ratio normalization compared to later initiation. Hence, the CD4/CD8 ratio might help to further discriminate the risk of disease progression of successfully treated HIV-infected individuals, and a successful response to ART may require both normalization of the peripheral CD4+ T cell count and the ratio of CD4+ to CD8+ T cell counts.

## Introduction

It is now anticipated that HIV-infected adults who have access to modern antiretroviral therapy (ART) should be able to suppress HIV replication indefinitely. Although treatment-mediated increases in the peripheral CD4 count are associated with reduced morbidity and mortality, compared to age-matched individuals without HIV infection, those on ART have a higher risk of morbidity and mortality. This risk is predicted in part by the on therapy CD4 count, although achieving an apparent normal CD4 count may not fully restore health [1]–[5]. Indeed, it has been shown that even those treated patients with CD4+ T cell counts above 500 cells/mm^3^, a further CD4+ T cell count increase is still associated with a slight benefit in terms of mortality [6]. The decreased life expectancy during ART-mediated viral suppression is largely explained by a higher than expected risk of non-AIDS-morbidity, a term that entails a group of conditions generally associated with aging, including cardiovascular, renal, liver, neurologic, and bone disease, as well as cancer [4], [7], [8].

While the mechanisms driving the increased burden of aging-associated disease in HIV-infected individuals are not fully understood, an emerging body of evidence suggests that persistent innate and adaptive immune dysfunction and/or activation are major risk factors [9]–[12]. Many of the immunologic abnormalities that persist during therapy are similar to those observed in the elderly, raising the hypothesis that age-associated decline in immune function (“immunosenescence”) contributes to disease progression and adverse outcomes [13]–[16]. Markers of innate immune activation [e.g. interleukin (IL)-6, high-sensitivity C reactive protein (hs-CRP) and soluble CD14 (sCD14)], coagulation (fibrinogen, D-dimers), bacterial translocation (lipopolysaccharide), and T cell activation (HLADR and CD38 co-expression) are elevated despite effective ART and associated with subsequent morbidity and mortality, even after adjustment for CD4+ T cell count [17]–[21]. Induction of indoleamine 2,3-dioxygenase-1 (IDO) in monocytes and dendritic cells occurs during HIV infection and has been associated with impairment of the mucosal immunity and the maintenance of a chronic inflammatory state [22]. Collectively, these observations strongly suggest that an underlying mechanism not captured by CD4+ T cell count and HIV replication might be contributing to disease progression.

The importance of CD4 counts as a strong predictor of opportunistic infections and non-AIDS events has been widely investigated, but little attention has been paid to the prognostic significance of CD8 counts. During untreated HIV infection, CD8 counts increase as CD4 counts decline [23]. During ART-mediated viral suppression, some individuals achieving CD4 counts above 500 cells/mm^3^ experience a simultaneous decline in CD8 counts, leading to normalization of the CD4/CD8 ratio. Others, however, maintain high levels of circulating CD8+ T cells, and hence a persistently low CD4/CD8 ratio [24]. Among elderly HIV-uninfected adults, inversion of the CD4/CD8 ratio (<1.0) predicts all-cause mortality and is considered part of the immunosenescent phenotype [25]–[29]. In the setting of untreated HIV infection, the CD4/CD8 ratio predicts time to AIDS [30] and is associated with pre-ART CD4 and CD8 counts [24], [31]. Among treated adults with spectrum of CD4+ T cell counts, the ratio appears to correlate with markers of T cell activation and senescence [32], [33] and with the risk of non-AIDS morbidity and mortality [34]–[36]. Whether this is true in those with normalized CD4+ T cell counts is unknown.

We hypothesized that among ART-treated HIV-infected individuals with CD4 counts ≥500 cells/mm^3^, expansion of CD8+ T cells, reflected as a low CD4/CD8 ratio, may identify individuals with persistent innate and adaptive immune activation at greater risk of serious non-AIDS events. Since early ART initiation has been shown to reduce levels of T cell activation, we hypothesized that earlier ART initiation might also accelerate the rate of CD4/CD8 ratio normalization.

## Methods

### Subjects

Study subjects were sampled from four cohorts and two clinical trials: 1) SCOPE: a clinic-based cohort of over 1500 chronically HIV-infected participants and HIV-uninfected controls in San Francisco; 2) the Study of the Ocular Complications of AIDS (SOCA): a multicenter cohort of over 2200 HIV-infected participants who initiated ART with an AIDS diagnosis; 3) OPTIONS: a clinic-based cohort of participants diagnosed during acute/early HIV infection, previously described [37]; 4) the Madrid cohort: a clinic-based cohort of 2400 ART-treated individuals, 130 of whom developed serious non-AIDS events. 5) the raltegravir (NCT00631449) [38] and maraviroc (NCT00735072) [39], [40] ART intensification randomized, placebo-controlled, clinical trials in HIV-infected individuals. Additional information on the cohorts and the clinical trials can be found in **Text S1**.

### Ethics statement

These studies were approved by the UCSF Committee on Human Research or by the Ethics Committee of the University Hospital Ramón y Cajal. All participants were adults and provided written informed consent in accordance with the Declaration of Helsinki.

### Measurements

T cell immunophenotyping was performed on cryopreserved peripheral blood mononuclear cells (PBMC), in fresh lymph node mononuclear cells (LNMC) from inguinal biopsies obtained from HIV-infected volunteers under ART-mediated viral suppression, and in mucosal mononuclear cells (MMC) obtained from rectal biopsies in the maraviroc and raltegravir studies, as previously described [38], [39], [41]. Fresh inguinal lymph nodes were biopsied were minced and strained through a 70 micron filter to created a single cell suspension of LNMC. MMC were isolated from biopsy specimens using a protocol optimized for lymphocyte viability and yield [42]. Cells were thawed, washed, stained with LIVE/DEAD Fixable Aqua Dead Cell Stain Kit (Invitrogen) to exclude non-viable cells and stained with fluorescently-conjugated monoclonal antibodies (recognizing CD3, CD4, CD8, HLADR, CD38, CD27, CD28, CCR5, CCR7, CD45, PD1 for PBMC and CD3, CD4 and CD8 for LNMC and MMC; see **Table S1**). Cells were then fixed in 0.5% formaldehyde and ≥250,000 were analyzed on a BD LSR II Flow cytometer (BD Biosciences) using FlowJo (Tree Star) to determine the proportion of CD4+ and CD8+ T cells expressing each of the T cell markers. Combinations of markers were calculated in FlowJo, using the Boolean gate function (for the gating strategy, see **Figure S1**). We determined in PBMC the T cell maturation subsets, defined as naïve (T_N_, CD45RA+CCR7+CD27+CD28+), central memory (T_CM_, CD45RA-CCR7+CD27+CD28+), transitional memory (T_TM_, CD45RA−CCR7−CD27+), effector memory (T_EM_, CD45RA−CCR7−CD27−CD28−), and terminally differentiated (T_EMRA_, CD45RA+CCR7−CD27−CD28−). For CD4+ T_TM_ we analyzed the CD28+ subset (CD45RA−CCR7−CD27+CD28+) and for CD8+ T_TM_ cells we analyzed the CD28− (CD45RA−CCR7−CD27+CD28−) given the strong correlation between the CD4/CD8 ratio and CD8+CD28− T cells. We also determined the phenotypes of activated/senescent CD8+ T cells (HLADR+CD38+, CD28−, CD57+CD28−, and PD-1+), and the proportion of CD28−CD8+ T cells expressing CD57, which has been recently described as a unique CD8+ T cell defect in HIV that appears to be distinct from the classical immunosenescent phenotype found with aging and that predicts mortality [43]. In LNMC and MMC we determined the % of CD4+ and CD8+ T cells. Additional information is provided in the supplemental material.

Cryopreserved plasma was assessed by immunoassay for IL-6 (R&D Systems), sCD14 (R&D Systems), hs-CRP (CardioPhase hs-CRP assay, Siemens), D-dimer (DiagnosticaStago), intestinal fatty acid binding protein (I-FABP, Cell Sciences) and zonulin-1 (ALPCO) levels. Plasma tryptophan and kynurenine levels were measured by high performance liquid chromatography tandem mass spectroscopy [22], and the activity of IDO was assessed as the plasma kynurenine to tryptophan (KT) ratio. Chronic asymptomatic CMV infection was confirmed by a positive CMV IgG titer and for a subset of HIV-infected participants without available CMV serology, >0.1% pp65/IE-specific IFN-γ+ CD8+ T cell responses by cytokine flow cytometry (ten-fold increase over limit of detection) as previously described [44].

### Statistical methods

Cross-sectional pairwise comparisons between groups were performed using Wilcoxon rank sum tests. Since a “normal” CD4/CD8 ratio remains poorly defined, for the between-group comparisons of T cell subsets and percentages of activated/senescent CD8+ T cells, we classified individuals according to the lowest quartile (≤0.4) and highest quartile (≥1.0) of SCOPE participants with ≥500 CD4+ T cells/mm^3^. A CD4/CD8 ratio ≤0.4 has been defined previously as the best cutoff that may predict serious non-AIDS events in well-treated HIV-infected patients [36], and 1.0 has been suggested in the general population as the cutoff for the “immune risk profile” associated with immunosenescence and mortality [26], [45]. To assess the intra-individual variability of the CD4/CD8 ratio, we used data from the control arms of ART intensification trials with raltegravir [38] and maraviroc [39], [40] to calculate the coefficient of variation (standard deviation/mean) for the CD4+ and CD8+ T cell counts and for the CD4/CD8 ratio.

To analyze the association between the CD4/CD8 ratio and the KT ratio in SOCA, we fitted a linear regression model using CD4/CD8 ratio as the dependent variable, and KT ratio as the explanatory variable, adjusting the model by age, gender, time under viral suppression and CD4 nadir. To evaluate the relative contribution of the CD4+ and CD8+ T cells to this association, we also fitted another model with both CD4+ and CD8+ T cells in which the CD4/CD8 ratio was not considered because of colinearity, adjusting for the same covariates.

We analyzed the correlations between the CD4/CD8 ratio in blood, with the ratio in lymph nodes and in GALT. For the GALT CD4/CD8 ratio measured in the MVC and RAL studies, we used only baseline measurements (before ART intensification). Since a different panel of antibodies was used for each study for flow-cytometry analysis, we fitted a linear regression analysis adjusting by the source study.

We analyzed the impact of early ART initiation on the CD4/CD8 ratio in the OPTIONS cohort among recently HIV-infected participants, focusing on those who either started ART within six months of infection (early ART) or who deferred therapy for at least two years (later ART) [37]. Longitudinal changes in CD4 and CD8 counts and in the CD4/CD8 ratio were assessed using linear mixed models with random intercepts. Age, gender, and pre-ART CD4 counts were included in multivariate analyses as fixed-effects. Interaction terms were created to assess whether these changes over time differed significantly between the early and later ART initiators. Changes in slopes before and after ART time points were assessed using linear splines.

We used data from the Madrid and SOCA cohorts to evaluate whether the CD4/CD8 ratio might be a marker of non-AIDS-related morbidity and mortality, respectively. In the nested case-control analysis in the Madrid cohort, cases who developed serious non-AIDS events and had ≥500 CD4+ T cells/mm^3^, were each matched to one controls by age, sex, nadir CD4, and proximal CD4 counts (N = 66). In the nested case-control study of immunological predictors of mortality in SOCA, cases with non-accidental death who had PBMC and plasma samples available within 18 months of death with confirmed plasma HIV RNA levels <400 copies/ml, were each matched to two controls by age, gender, duration of viral suppression, history of CMV retinitis, and nadir CD4 (N = 183). We used conditional logistic regression to evaluate the CD4/CD8 ratio as a predictor of non-AIDS morbidity/mortality. Continuous variables in multivariate models were log-transformed when necessary to satisfy model assumptions.

## Results

### Low CD4/CD8 ratio during effective ART is associated with T cell activation and prominent immunosenescence

We first analyzed the correlations of the CD4/CD8 ratio with naïve T cells (T_N_, CD45RA+CCR7+CD27+CD28+), central memory T cells (T_CM_, CD45RA−CCR7+CD27+CD28+), transitional memory T cells (T_TM_, CD45RA−CCR7−CD27+CD28+ for CD4+ cells and CD45RA−CCR7−CD27+CD28− for CD8+ cells), effector memory T cells (T_EM_, CD45RA−CCR7−CD27−CD28−), terminally differentiated T cells (T_EMRA_, CD45RA+CCR7−CD27−CD28−) and different phenotypes of activated T cells (HLADR+CD38+, CD28−, CD57+CD28−, and PD-1+). All data were obtained from those participants in SCOPE cohort who were on effective therapy and had ≥500 CD4+ T cells/mm^3^ (N = 67) (for a description of the general characteristics, see **Table S2**). The CD4/CD8 ratio was correlated positively with the frequencies of T_N_ (Rho = 0.35, P = 0.005), T_CM_ (Rho = 0.272, P = 0.03), and T_TM_ CD8+ T cells (Rho = 0.25, P = 0.05), and negatively with the frequencies of T_EM_ (Rho = −0.37, P = 0.003) and T_EMRA_ (Rho = −0.26, P = 0.024) CD8+ T cells. Overall, the CD4/CD8 ratio was more strongly associated with the proportions of T cell maturation subsets and proportions of activated CD8+ T cell phenotypes than were the CD4 or CD8 counts (see **Table 1**).

**Table 1:** CD4+ T cell counts, CD8+ T cell counts and CD4/CD8 ratio correlations with the percentage of T cell maturation subsets and T cell activation phenotypes in HIV-infected participants in SCOPE cohort.

	CD4+ T cell count Rho (P value)	CD8+ T cell count Rho (P value)	CD4/CD8 ratio Rho (P value)
**ALL SUBJECTS (n = 95)**			
**%CD4+ T cells**			
***Maturational subsets***			
**Naïve**	**0.395 (<0.001)**	−0.027 (0.798)	**0.329 (0.001)**
**T_CM_**	−0.047 (0.656)	−0.069 (0.511)	−0.019 (0.857)
**T_TM_**	**−0.194 (0.065)**	0.038 (0.720)	**−0.179 (0.090)**
**T_EM_**	**−0.366 (0.004)**	−0.092 (0.931)	**−0.219 (0.036)**
**T_EMRA_**	−0.051 (0.633)	−0.077(0.468)	0.021 (0.837)
***Activation phenotypes***			
**HLADR+CD38+**	**−0.577 (<0.001)**	0.008 (0.937)	**−0.410 (<0.001)**
**CD28−CD57+**	**−0.209 (0.048)**	−0.004 (0.968)	−0.149 (0.159)
**PD1+**	**−0.565 (<0.001)**	−0.037 (0.731)	**−0.375 (<0.001)**
**%CD8+ T cells**			
***Maturational subsets***			
**Naïve**	**0.324 (0.002)**	**−0.252 (0.016)**	**0.437 (<0.001)**
**T_CM_**	0.011 (0.918)	−0.159 (0.131)	0.123 (0.245)
**T_TM_**	0.037 (0.727)	**0.239 (0.023)**	**0.203 (0.053)**
**T_EM_**	−0.167 (0.106)	**0.319 (0.002)**	**−0.379 (<0.001)**
**T_EMRA_**	−0.185 (0.079)	0.167 (0.112)	**−0.297 (0.004)**
***Activation Phenotypes***			
**HLADR+CD38+**	**−0.301 (0.003)**	−0.159 (0.133)	**−0.324 (0.002)**
**CD28−CD57+**	−0.022 (0.838)	0.180 (0.088)	−0.156 (0.140)
**CD28−**	−0.234 (0.026)	**0.268 (0.010)**	**−0.381 (<0.001)**
**%CD57+of CD28−**	0.177 (0.094)	−0.082 (0.441)	0.203 (0.054)
**PD1+**	−0.029 (0.787)	−0.022 (0.831)	0.026 (0.807)
**SUBJECTS WITH CD4≥500 cells/mm^3^**			
**%CD4+ T cells**			
***Maturational subsets***			
**Naïve**	−0.026 (0.842)	−0.109 (0.397)	0.109 (0.394)
**T_CM_**	0.035 (0.787)	−0.138 (0.279)	0.134 (0.292)
**T_TM_**	0.036 (0.779)	0.069 (0.589)	−0.019 (0.879)
**T_EM_**	−0.088 (0.495)	0.005 (0.684)	−0.101 (0.431)
**T_EMRA_**	0.087 (0.499)	−0.033 (0.799)	0.011 (0.927)
***Activation phenotypes***			
**HLADR+CD38+**	−0.171 (0.179)	0.113 (0.380)	−0.148 (0.247)
**CD28−CD57+**	0.098 (0.450)	0.071 (0.582)	−0.062 (0.633)
**PD1+**	−0.133 (0.299)	−0.052 (0.685)	0.016 (0.890)
**%CD8+ T cells**			
***Maturational subsets***			
**Naïve**	−0.053 (0.681)	**−0.383 (0.002)**	**0.347 (0.005)**
**T_CM_**	0.041 (0.744)	**−0.264 (0.004)**	**0.272 (0.031)**
**T_TM_**	−0.009 (0.947)	0.237 (0.061)	−0.192 (0.131)
**T_EM_**	−0.032 (0.803)	**0.376 (0.002)**	**−0.372 (0.003)**
**T_EMRA_**	−0.104 (0.419)	**0.319 (0.011)**	**−0.285 (0.023)**
***Activation Phenotypes***			
**HLADR+CD38+**	−0.031 (0.773)	**0.273 (0.031)**	−0.234 (0.062)
**CD28−CD57+**	0.048 (0.7112)	**0.362 (0.004)**	**−0.323 (0.009)**
**CD28−**	−0.002 (0.986)	**0.453 (0.002)**	**−0.426 (<0.001)**
**%CD57+of CD28−**	0.096 (0.455)	0.001 (0.995)	0.028 (0.828)
**PD1+**	−0.002 (0.984)	−0.105 (0.411)	0.148 (0.284)

“%CD57 of CD28-” refers to the percentage of CD28−CD8+ T cells expressing CD57.

**Figure 1:**
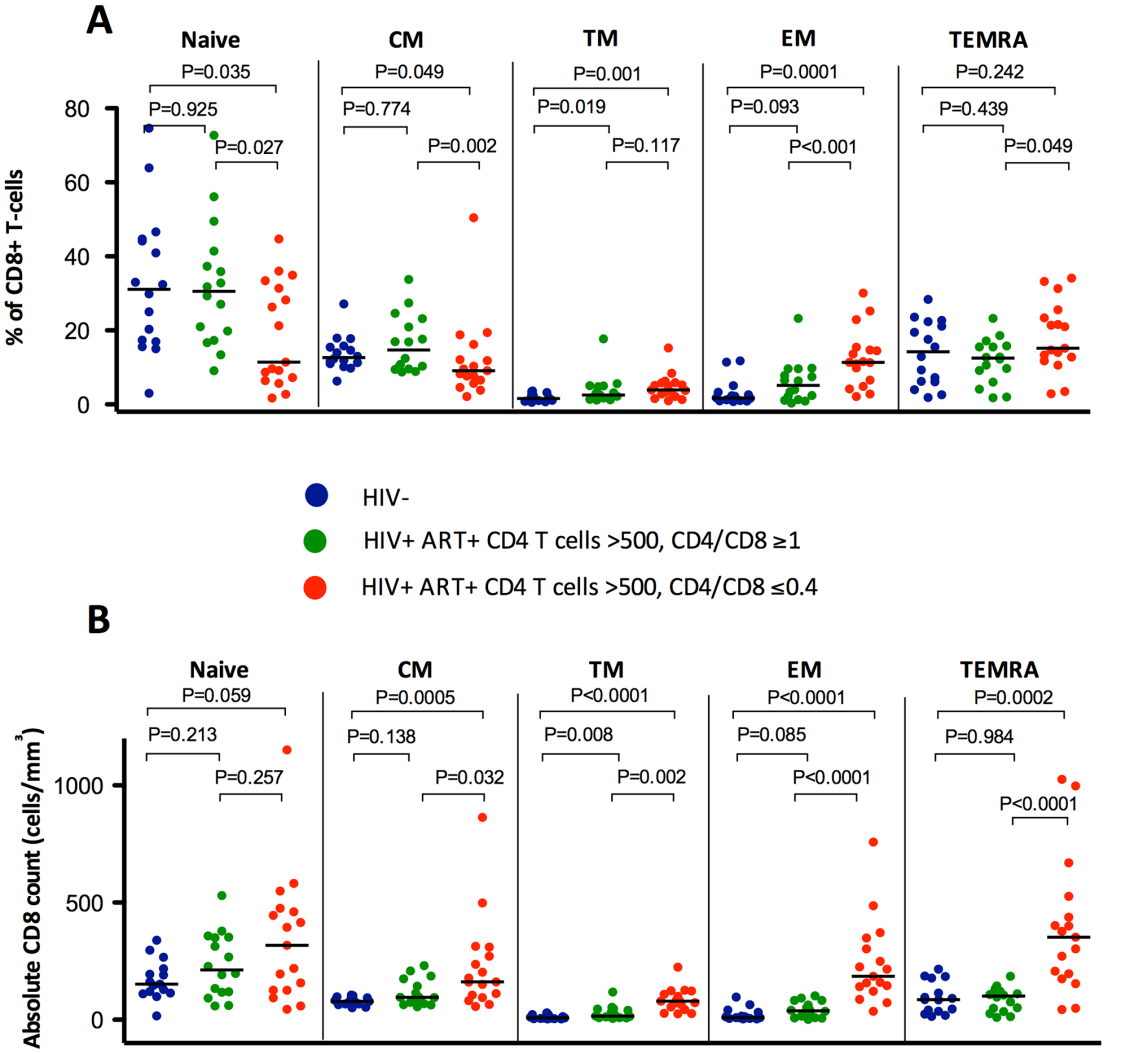
Percentages and absolute counts of CD8+ T cell maturation subsets among HIV-/CMV+ individuals and ART-suppressed HIV-infected patients with CD4 counts >500 cells/mm^3^ stratified by a normal (4th quartile, ≥1) or low (1st quartile, ≤0.4) CD4/CD8 ratio. HIV-infected individuals with low CD4/CD8 ratio had lower percentages of T_N_, T_CM_, and T_TR_ CD8+ cells, higher T_EM_ and T_EMRA_ (**A**), and higher absolute counts (**B**) of all subsets compared to those with higher CD4/CD8 ratio and with healthy controls.

**Figure 2:**
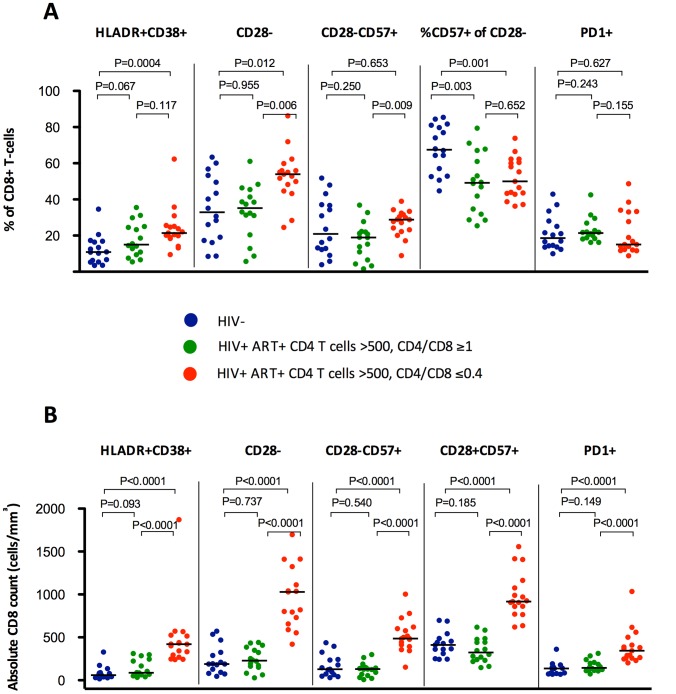
Percentages and absolute counts of CD8+ activation phenotypes among HIV-/CMV+ individuals and ART-suppressed HIV-infected patients with CD4 counts >500 cells/mm^3^ stratified by a normal (4th quartile, ≥1, in green) or low (1st quartile, ≤0.4, in red) CD4/CD8 ratio. Subjects with low CD4/CD8 ratio showed higher percentages (**A**) and absolute counts (**B**) of HLADR+, CD28− and CD28−CD57+, and higher absolute counts of PD1+ cells (**B**). There were no differences in HIV-infected individuals in the proportion of CD28−CD8+ T cells expressing CD57, being significantly lower in both groups compared to HIV-/CMV+ controls.

To underline the association between a low CD4/CD8 ratio and persistent T cell abnormalities during effective ART, we compared ART-suppressed HIV-infected individuals with ≥500 CD4+ T cells/mm^3^ (N = 67) in the lowest (≤0.4) versus highest (≥1) quartiles of CD4/CD8 ratio. We also analyzed HIV-uninfected CMV-positive adults (N = 15) (see **Table S2** for the clinical characteristics of each group and **Figures 1** **,** **2** **and S2** for the between-group comparisons). Median CD8 counts were markedly higher among those with low versus high CD4/CD8 ratio (1964 cells/mm^3^ vs. 696 cells/mm^3^, respectively). ART-suppressed participants with a high CD4/CD8 ratio had similar proportions of CD8+ T cell maturation subsets as those in the healthy controls (**Figure 1A–B**). In contrast, those participants with a low ratio had higher frequencies of T_TM_ and T_EM_ CD8+ T cell subsets than that observed in the healthy controls. Compared to HIV-uninfected subjects, ART-suppressed individuals with low CD4/CD8 ratio had higher proportions of activated (HLADR+CD38+) and “senescent” (CD28− and CD28−CD57+) CD8+ T cells; while those with high CD4/CD8 ratio had levels of CD8+ T cell activation and senescence close to those observed in controls. However, both ART-suppressed groups (low and high CD4/CD8 ratios) had lower proportions of CD28−CD8+ T cells expressing CD57 compared to healthy controls, consistent with prior data (**Figure 2A**) [43].

We sought to validate these findings among effectively treated subjects (undetectable viral load, ≥500 CD4+ T cells/mm^3^) within the SOCA cohort (general characteristics summarized in **Table S3**), and found comparable correlations between the CD4/CD8 ratio and different phenotypes of activated/senescent CD8+ T cells among ART-suppressed subjects with CD4>500 T cells/mm^3^. The most consistent correlates of the CD4/CD8 ratio were the %HLADR+CD38+ CD8+ T cells (Rho = −0.507, P<0.001) and %CD28− CD8+ T cells (Rho = −0.400, P = 0.009) (**Table 2**).

**Table 2:** Correlations with biomarkers of T cell activation and senescence in SOCA cohort.

All subjects (n = 192)	CD4 Rho (P value)	CD8 Rho (P value)	CD4/CD8 ratio Rho (P value)
**%CD8+ T cells**			
**HLADR+CD38+**	**−0.425 (<0.001)**	−0.014 (0.862)	**−0.394 (<0.001)**
**CD28−CD57+**	**−0.177 (0.027)**	−0.012 (0.878)	**−0.213 (0.007)**
**CD28−**	**−0.241 (0.002)**	−0.006 (0.428)	**−0.323 (<0.001)**
**%CD57+of CD28−**	−0.028 (0.729)	−0.047 (0.563)	−0.011 (0.894)
**PD1+**	**−0.230 (0.038)**	0.005 (0.952)	**−0.267 (<0.001)**
**Subjects with CD4≥500 cells/mm^3^ (n = 49)**			
**%CD8+ T cells**			
**HLADR+CD38+**	−0.247 (0.115)	**0.425 (0.005)**	**−0.507 (<0.001)**
**CD28−CD57+**	−0.094 (0.552)	0.273 (0.080)	**−0.319 (0.040)**
**CD28−**	−0.122 (0.441)	0.348 (0.023)	**−0.400 (0.009)**
**%CD57+of CD28−**	0.043 (0.785)	0.172 (0.276)	−0.142 (0.368)
**PD1+**	−0.287 (0.065)	0.222 (0.157)	−0.256 (0.102)

“%CD57 of CD28−” refers to the percentage of CD28−CD8+ T cells expressing CD57.

### Correlations between the CD4/CD8 ratio and markers of innate immune dysfunction

**Figure 3:**
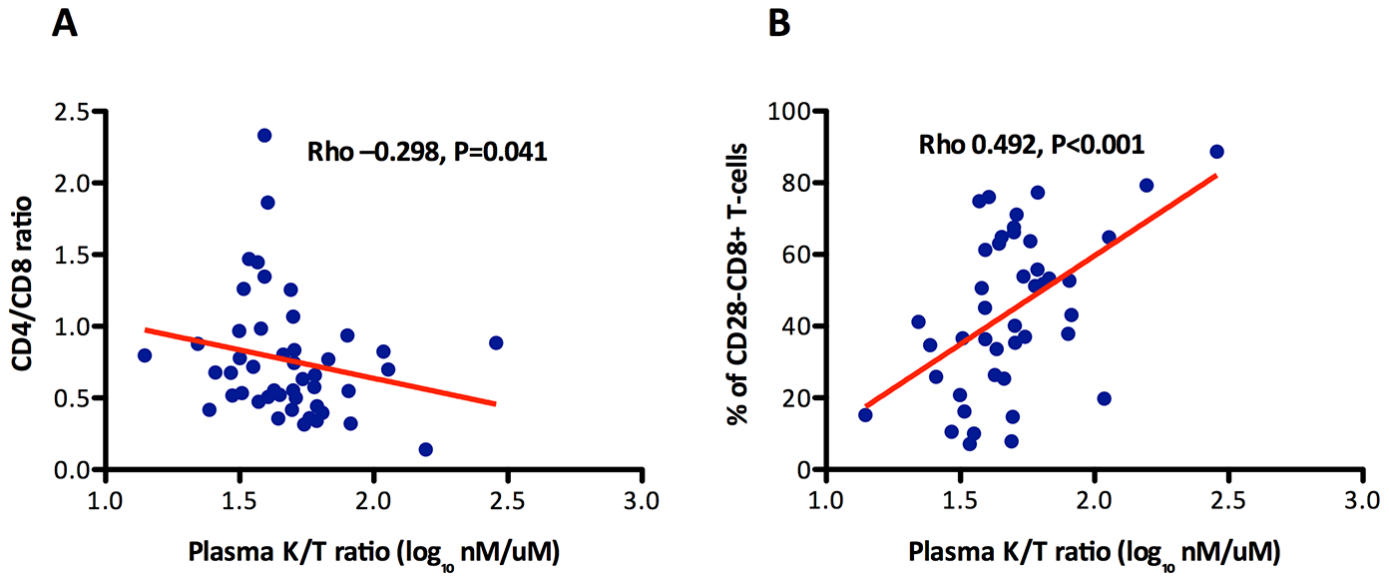
Association between the CD4/CD8 ratio and the % of CD28−CD8+ T cells with indoleamine 2,3-dioxygenase-1 (IDO-1) activity (kinurenine/tryptophan ratio) among participants in the SOCA cohort with 500 CD4+ T cells/mm^3^. The KT ratio significantly correlated with the CD4/CD8 ratio and the % of CD28+CD8+ T cells. The between the CD4/CD8 ratio and the KT ratio was confirmed in a linear regression analysis adjusted by age, gender and cumulative ART exposure (Beta = −0.72, P = 0.009). The red line represents a linear prediction.

To explore the potential mechanisms driving the expansion of late-memory T cells in subjects with low CD4/CD8 ratio despite effective ART we used data from the SOCA cohort. We analyzed the correlations between CD4 and CD8 counts and the CD4/CD8 ratio and different markers of innate immune activation and epithelial integrity (**Table 3**). We observed across all effectively treated subjects (as defined by having an undetectable viral load) significant inverse correlations between the CD4/CD8 ratio and hs-CRP, IL-6, sCD14 and the KT ratio. However, in the subgroup of subjects with ≥500 CD4+ T cells/mm^3^, only the KT ratio remained significantly correlated with the CD4/CD8 ratio (Rho = −0.30, P = 0.041) (**Figure 3** ***A***). This association was confirmed in a linear regression analysis adjusted for age, gender and cumulative ART exposure (Beta = −0.72, P = 0.009), where for each 10% increase in the CD4/CD8 ratio there was a 7% decrease in the KT ratio. The CD4/CD8 ratio performed better as a predictor of the KT ratio than the CD4+ or CD8+ T cell counts in a similar model (see **Table 4**). Since subjects with low ratio and ≥500 CD4+ T cells/mm^3^ were enriched for CD28−CD8+ T cells and also showed increased IDO induction, we hypothesized that a potential underlying mechanism driving expansion of CD28−CD8+ T cells might be IDO induction, and we found a positive correlation between these two variables (Rho = 0.50, P<0.001) (**Figure 3** ***B***). These results indicate that the CD4/CD8 ratio predicts better the degree of IDO induction than the CD4 or CD8 counts individually, which becomes especially evident above the threshold of 500 CD4+ T cells/mm^3^, and that in subjects with low CD4/CD8 ratio increased IDO induction is associated with an immunosenescent phenotype (expansion of CD28−CD8+ T cells).

**Table 3:** Correlations with biomarkers of innate immune activation and epithelial integrity in SOCA cohort.

All subjects (n = 192)	CD4 Rho (P value)	CD8 Rho (P value)	CD4/CD8 ratio Rho (P value)
**KT ratio**	**−0.225 (0.003)**	0.127 (0.095)	**−0.336 (<0.001)**
**sCD14**	**−0.228 (0.002)**	0.028 (0.708)	**−0.232 (0.002)**
**hs-CRP**	**−0.148 (0.049)**	−0.071 (0.345)	**−0.152 (0.043)**
**D-dimers**	−0.139 (0.063)	−0.05 (0.469)	−0.141 (0.060)
**Interleukin-6**	**−0.158 (0.035)**	−0.035 (0.639)	**−0.162 (0.031)**
**IFABP**	−0.138 (0.067)	0.015 (0.841)	−0.146 (0.052)
**Zonulin**	0.111 (0.142)	−0.048 (0.523)	0.108 (0.155)
**Subjects with CD4≥500 cells/mm^3^ (n = 49)**			
**KT ratio**	−0.209 (0.163)	0.252 (0.091)	**−0.298 (0.041)**
**sCD14**	**−0.394 (0.007)**	−0.011 (0.943)	−0.155 (0.303)
**hs-CRP**	−0.063 (0.673)	−0.023 (0.876)	−0.039 (0.799)
**D-dimers**	−0.004 (0.980)	−0.095 (0.537)	0.015 (0.919)
**Interleukin-6**	−0.152 (0.313)	−0.097 (0.521)	−0.012 (0.930)
**IFABP**	−0.144 (0.337)	0.144 (0.338)	−0.223 (0.134)
**Zonulin**	0.066 (0.664)	−0.048 (0.526)	−0.087 (0.569)

**Table 4:** Multivariate linear regression analysis: Associations of the KT ratio (dependent variable) with CD4+ and CD8+ T cells, and the CD4/CD8 ratio (independent variables) in SOCA cohort.

	Beta	Std. error	P value
**CD4+ T cells**			
All subjects	−0.366	0.095	<0.001
Subjects with CD4≥500 cells/mm^3^	−0.861	0.779	0.276
**CD8+ T cells**			
All subjects*	0.187	0.065	0.005
Subjects with CD4≥500 cells/mm^3^	0.279	0.149	0.053
**CD4/CD8 ratio**			
All subjects*	−0.374	0.094	<0.001
Subjects with CD4≥500 cells/mm^3^	−0.723	0.262	0.009

Because of colinearity, we fitted one model to calculate the coefficients of CD4+ and CD8+ T cells, and a different model for the CD4/CD8 ratio.

Variables CD4+ and CD8+ T cells, and the CD4/CD8 ratio were log transformed.

Coefficients are adjusted by age, gender, nadir CD4+ T cell count and duration of viral suppression.

To interpret the logarithmically transformed coefficients, we applied the following formula: Beta*log(1.10), resulting in the % of change in the odds of the outcome predicted by each 10% increase in the independent variable.

### Correlations between the CD4/CD8 ratio in blood and lymphoid tissues

**Figure 4:**
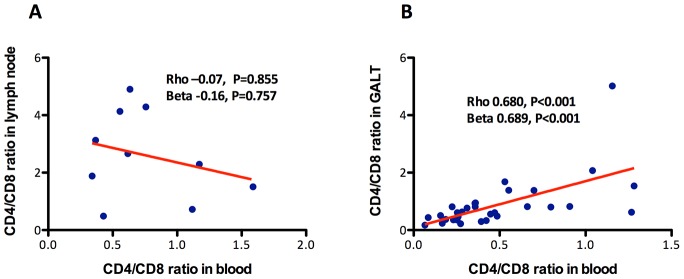
Association between the CD4/CD8 ratio in blood and in lymph nodes or in GALT. While no association with the CD4/CD8 ratio in lymph nodes was detected (**A**), it correlated positively with the ratio in GALT (**B**). The red line represents a linear prediction.

A persistently low CD4/CD8 count ratio in peripheral blood might conceivably be the result of differential redistribution of CD4+ and CD8+ T cells out of lymphoid tissues, but to our knowledge, no study has assessed whether the CD4/CD8 ratio in peripheral blood is reflective of the CD4/CD8 ratio in tissues. To address this question, we analyzed the correlations of the CD4/CD8 ratio in blood with the CD4/CD8 ratio in lymph node and in GALT (see **Table S4**). Using data from 10 individuals on ART, in whom the CD4/CD8 ratio in blood was 0.6 (0.4–1.1) and in lymph nodes 2.5 (1.5–4.1), no significant correlation between the CD4/CD8 ratio in blood and in lymph nodes was detected (Rho = −0.07, P = 0.855) (**Figure 4** ***A***). For the correlation between the CD4/CD8 ratio in GALT and in blood (**Figure 4** ***B***), we used data from 32 individuals on ART, in whom the CD4/CD8 ratio in blood was 0.4 (0.2–0.6) and in GALT 0.6 (0.4–0.9). We found that the CD4/CD8 ratio in blood was strongly correlated with that in rectal mucosa (Rho = 0.68, P<0.001 and Beta = 0.69, P<0.001).

### Lower intra-individual variability of the CD4/CD8 ratio compared to CD4+ and CD8+ T cell counts

To characterize the variability on CD4/CD8 ratios over time, we analyzed data from 38 HIV-infected adults who maintained undetectable viral loads on ART. Subjects had a median CD4+ count of 320 cells/mm^3^ (range 88 to 884) at baseline. A median of 11 determinations of CD4+ and CD8+ T cells measurements were performed during a median of 81 weeks (**Figure S3**). The mean coefficient of variation was significantly lower for the CD4/CD8 ratio (12%) compared to CD4+ T cell counts (16%, P = 0.017) and for CD8+ T cell counts (18%, P = 0.001), indicating that the CD4/CD8 ratio shows lower intra-individual than the CD4+ or CD8+ T cell counts over time.

### Impact of timing of ART initiation on the dynamics of circulating CD4+ T cells, CD8+ T cells and CD4/CD8 ratio

**Figure 5:**
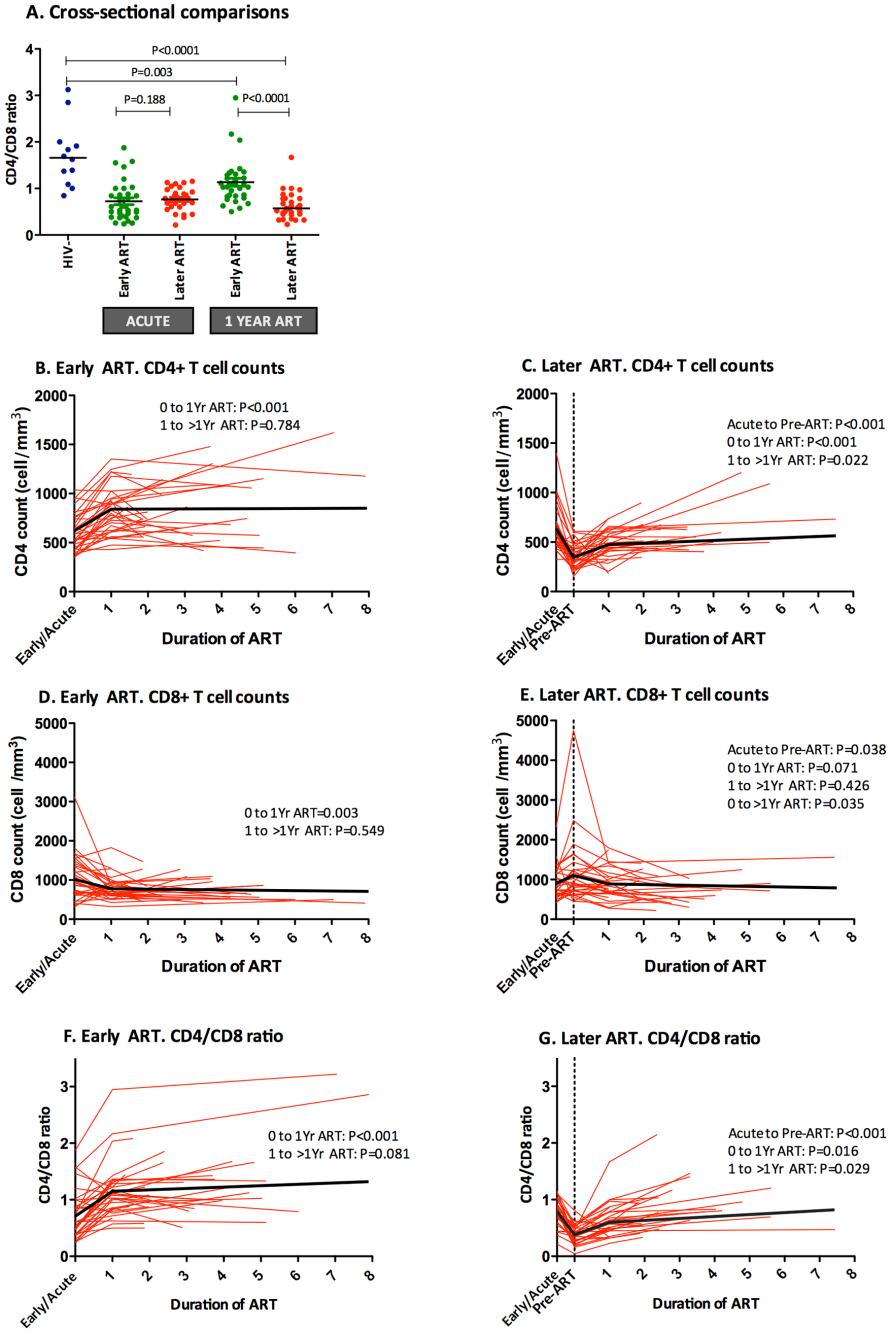
Impact of early or later ART initiation in peripheral CD4+ T cell counts, CD8+ T cell counts and CD4/CD8 ratio in the OPTIONS cohort. The CD4/CD8 ratio was compared between HIV-uninfected individuals (blue) and HIV-infected individuals initiating ART “early,” ≤6 months of infection (green), or “later,” ≥2 years after initial infection (red), at acute HIV diagnosis and after 1 year of ART. Median CD4/CD8 ratio was significantly higher after one year in early ART initiators compared to later initiators. (**A**). Early ART initiators experienced higher CD4+ T cell increase (**B**) than later initiators (**C**) after one year of ART (221 cells/mm^3^ vs. 130, respectively, P<0.001). No differences were observed in CD8+ T cell counts between early (**D**) and later ART initiators (**E**) after one year of ART (−212 cells/mm^3^ vs. −114, respectively, P = 0.098) but CD8+ T cells were significantly different between groups beyond one year of ART (−309 cells/mm^3^ vs. −114, respectively, P = 0.014). Changes in the CD4/CD8 ratio among recently HIV-infected individuals initiating ART early (**F**) and later (**G**) were also assessed over time. Early ART initiators experienced a higher increase at one year of ART than later initiators (+0.43 vs. +0.25, P<0.001). Individual participant trajectories shown with red lines, estimated mean values over time from linear mixed models adjusted by age, sex, baseline CD4+ T cells shown in thick black lines.

We next examined the extent to which persistent abnormalities in the CD4/CD8 ratio were associated with later vs. earlier initiation of ART using the described OPTIONS cohort of recently infected adults who started therapy during the first six months of their infection (early ART) or after two years of untreated infection (later ART) (see **Table S5**) [37]. At the time of their diagnosis, median CD4/CD8 ratio was significantly lower in recently HIV-infected individuals compared to the HIV-uninfected group (**Figure 5** ***A***, all baseline comparisons, P<0.05). The later ART group remained untreated for a median of 3 years. The CD4 count declined by 274 cells/mm^3^, the median CD8 count increased by 125 cells/mm^3^, and the CD4/CD8 ratio decreased from 0.76 to 0.38 during this untreated period.

After one year of ART, both early and late ART initiators experienced a substantial increase in CD4+ T cells (**Figures 5** ***B–C***). However, while early ART subjects also showed a substantial decline in CD8+ T cells after one year of ART, the later ART group required a median follow-up of three years (**Figures 5** ***D–E***). After one year of ART, early treated patients showed significantly higher median CD4/CD8 ratio (1.0 vs. 0.57, P<0.001) and had fourfold-increased odds of CD4/CD8 ratio normalization during follow-up (OR, 3.6; 95% CI, 1.2, 10.8; P = 0.022). The greater effect of early ART compared to later ART on the CD4/CD8 ratio remained statistically significant after adjustment by age, gender, and baseline CD4+ T cell counts in the mixed-effects linear model (**Figures 5** ***F–G***). The mean CD4/CD8 ratio change predicted by the model was significantly higher among early ART initiators compared to later initiators after one year of ART (+0.44 vs. +0.25, respectively, P<0.001), and after a median of 3 years of ART (+0.61 vs. +0.49, respectively, P<0.001). In summary, early ART initiators experienced a faster CD4/CD8 ratio increase and reached higher CD4/CD8 ratios after a median of 3 years of ART, which was primarily driven by changes in the CD4 counts and, to a lesser degree, by changes in the CD8 counts.

### Low CD4/CD8 ratio despite effective ART predicted disease progression beyond the CD4+ and CD8+ T cell counts

Lastly, we hypothesized that the prognostic importance of the CD4/CD8 ratio might depend upon the relative predictive contribution of both CD4 and CD8 counts. In a previously reported analysis, we found that among a large cohort of treated adults in Madrid (N = 420) that the ratio was predictive disease progression [36]. Here, we performed a case-control study among the subset with ≥500 cells/mm^3^. A sample of 33 cases with CD4 counts ≥500 cells/mm^3^ was matched to 33 controls by age, gender, nadir CD4 and proximal CD4+ T cell counts (see **Table S6** for the general characteristics of the study population and **Table S7** for the description of non-AIDS events). We observed that both the CD4/CD8 ratio and CD8 count independently predicted the risk of non-AIDS events, with the coefficient of the CD4/CD8 ratio significantly higher (**see** **Table 5**). After controlling for age, gender, ART duration, nadir and proximal CD4 count, each 10% decrease in the CD4/CD8 ratio and each 10% increase in the CD8+ T cell counts were associated with 48% and 22% higher odds of serious non-AIDS events, respectively.

**Table 5:** Conditional logistic regression analysis: Predicted morbidity and mortality by the CD4+ and CD8+ T cell counts and the CD4/CD8 ratio in the Madrid cohort and SOCA cohort mortality nested studies.

	Beta	Std. error	P value
**Madrid cohort (N = 66) (all subjects CD4≥500 cells/mm^3^)**			
**CD4+ T cells**			
Unadjusted	−1.86	2.85	0.514
Adjusted by ART duration	−0.66	3.76	0.859
**CD8+ T cells**			
Unadjusted	2.80	1.12	0.013
Adjusted by ART duration	2.29	1.16	0.048
**CD4/CD8 ratio**			
Unadjusted	−6.23	2.48	0.012
Adjusted by ART duration	−5.08	2.53	0.045
**SOCA cohort (N = 192)**			
**CD4+ T cells**			
All subjects	−1.52	0.58	0.009
Subjects with CD4≥500 cells/mm^3^ *	−4.09	6.43	0.525
**CD8+ T cells**			
All subjects*	0.28	0.33	0.392
Subjects with CD4≥500 cells/mm^3^ *	2.37	2.05	0.246
**CD4/CD8 ratio**			
All subjects*	−1.38	0.55	0.012
Subjects with CD4≥500 cells/mm^3^ *	−5.04	3.88	0.194

*N = 47.

Because of colinearity, we fitted one model to calculate the coefficients of CD4+ and CD8+ T cells, and a different model for the CD4/CD8 ratio.

Variables CD4+ and CD8+ T cells, and the CD4/CD8 ratio were log transformed.

Coefficients are adjusted by age, gender, nadir CD4+ T cell count and duration of viral suppression.

To interpret the logarithmically transformed coefficients, we applied the following formula: Beta*log(1.10), resulting in the % of change in the odds of the outcome predicted by each 10% increase in the independent variable.

To assess the relationships with mortality, we also examined the entire SOCA cohort (median CD4+ T cell count at baseline of 340 cells/mm^3^, range 1 to 1498). We analyzed 62 individuals who died (cases) and 121 who did not die (controls) matched by age, gender, nadir CD4+ T cell count, and duration of viral suppression (see **Tables S3** and **S7**). We observed that both the CD4/CD8 ratio and CD4+ T cells, but not CD8+ T cells, were independent predictors of mortality –for each 10% increase in the CD4/CD8 ratio or in CD4+ T cells and there was a 15% and 13% decrease in the risk of death, respectively (**see** **Table 5**). As this cohort enrolled individuals with advanced HIV disease, there were insufficient numbers of events in the ≥500 cells/mm^3^ subset to analyze.

## Discussion

Combining the data from four clinical cohorts and two clinical trials, we demonstrate here that a substantial subset of ART-suppressed HIV-infected adults who have achieved virologic suppression and a normalized peripheral CD4 count (≥500 cells/mm^3^) have persistently elevated CD8 counts and a low CD4/CD8 ratio. This ratio is correlated with markers of T cell activation and innate immune activation (IDO induction) and with the presence of a previously described immunosenescent phenotype (i.e., low naïve T cell frequencies and increased frequency of terminally differentiated). This imbalance in T cell homeostasis measured in blood is also present in GALT, and the CD4/CD8 ratio shows lower intra-individual variability than the CD4+ or CD8+ T cell counts. Although early ART (<6 month after HIV infection) is associated with more rapid normalization of the CD4/CD8 ratio, an abnormal ratio persists even in these aggressively treated individuals. Among well-treated individuals with high CD4 count, a low ratio was an independent predictor of serious non-AIDS events and mortality. Collectively, these results suggest that a persistently low CD4/CD8 ratio during ART may be a marker of persistent immune dysfunction and inflammation, and that monitoring of this ratio—which can be readily done in most clinics with current assays—may be clinically useful. A truly successful response to ART may require both normalization of the peripheral CD4 count and the CD4/CD8 ratio.

The immunologic profile of the individuals in our cohorts with a persistently low CD4/CD8 ratio despite high CD4 counts is similar to that observed in the very old. T cell “immunosenescence” is generally defined as a low naïve/memory T cell ratio, expansion of CMV-specific CD8+ T cells, enrichment for CD28− and PD-1+ T cells, increased CRP and IL-6 levels, reduced T cell telomere lengths and a low CD4/CD8 ratio [46]. Since untreated HIV infection is associated with each of these immunologic characteristics, it has been proposed that HIV might accelerate the aging of human immune system [13], [47]–[49]. The extent to which successful ART reverses these HIV-induced immune changes is currently the subject of intense investigation [31]. We found that CD8+ T cells counts often remain high even as CD4+ T cell count levels normalize, arguing against the existence of a “blinded” T cell homeostasis during long-term ART. We also found that among apparently well-treated adults (undetectable viral load, high CD4+ T cell counts) that a persistently low CD4/CD8 ratio was consistently associated with markers of inflammation (particularly CD8+ T cell activation).

We also studied a number of related markers of immunosenescence, using as a comparator group HIV-uninfected adults who were infected with CMV (as nearly all HIV-infected subjects are co-infected with this virus). We found that HIV-infected subjects who achieved CD4/CD8 ratio normalization during ART demonstrated traits of a nearly healthy immune system, with T cell maturation subsets and levels of CD8+ T cell activation/senescence comparable to those observed in healthy subjects. In contrast, a CD4/CD8 below ≤0.4 identified individuals with prominent features of immunosenescence despite CD4+ T cell recovery, including reduction of the CD8+ naïve T cell compartment, enrichment for T_EM_ and T_EMRA_ CD8+ cells, and increased levels of CD8+ T cell activation (HLADR+CD38+ T cells) and senescence (CD28− and CD57+CD28− T cells). Expansion of CD28−CD8+ T cells is a hallmark of replicative senescence, a term describing the phenomenon in which long-lived cells that have undergone multiple rounds of proliferation, show telomere shortening and hence, limited proliferative potential [50],[51].

When studied in all treated subjects, the CD4/CD8 ratio inversely correlated with several markers of innate immune activation (sCD14, hs-CRP, and IL-6) and with a biomarker of IDO induction (KT ratio). While the association with the KT ratio remained significant in the subgroup of individuals with ≥500 CD4+ T cells/mm^3^, the correlations for sCD14, hs-CRP, and IL-6 did not, perhaps because of the very high biologic variability in these assays and the loss of statistical power in the subgroup analysis. IDO is induced during HIV infection in activated dendritic cells and monocytes by interferons and toll-like receptors ligands such as LPS and sCD14, catabolizing tryptophan into kynurenine and other immunologically active catabolites; these catabolites suppress T cell proliferation and/or differentiation, resulting in impairment of the mucosal immunity. Such induction may occur even after the initiation of effective ART, as shown by increased K/T ratios in some if not all [52]. It has been argued that induction of IDO may represent a critical initiating event that results in inversion of the Treg/TH17 regulatory balance, loss of epithelial barrier integrity and thereby maintenance of a chronic inflammatory state during chronic HIV infection [22], [53]. Since the KT ratio correlated well with CD28−CD8+ T cells, our data suggest that increased IDO activity may be contributing to the replicative CD8+ T cell senescence observed in ART-treated subjects with low CD4/CD8 ratio. Alternatively, the accumulation of CD8+ T cells might be the cause of increased IDO activity, as a consequence of greater IFN-γ production.

We observed that a low CD4/CD8 ratio is a predictor of serious non-AIDS events among treated individuals with ≥500 CD4+ T cells/mm^3^ in the Madrid-based cohort, with much of the association driven by the CD8 counts. This observation expands upon our previous findings in this cohort, where we showed in a population of HIV-infected individuals non-restricted by a CD4 count that a low ratio is associated with increased risk of non-AIDS events and associated mortality [36]. We observed an association between the ratio and mortality in the entire SOCA cohort, but in this cohort selected based on low CD4 nadir and which had a lower range of CD4 counts, the association depended upon the CD4 counts. These two analyses suggest that while the CD4/CD8 ratio has prognostic significance in all treated adults, much of the harm associated with a low ratio is driven by the CD4 count (and presumably immunodeficiency) in those with low CD4+ T cell counts, while the harm associated with low ratio in those with higher CD4+ T cell counts is driven by CD8+ T cell count (and presumably inflammation).

As suggested by the present study, the group of ART-treated HIV-infected individuals with CD4+ T cell counts above 500 cells/mm^3^ represents a clinical spectrum of individuals, ranging from those with an immune system that is abnormal (e.g., with a CD4/CD8 ratio ≤0.4) to those with an apparently normal immune system (e.g., with a CD4/CD8 ratio ≥1), a finding that might serve to explain the discrepancies in cohort studies addressing morbidity and mortality among successfully treated HIV-infected individuals [4], [6]–[8], [54]–[57]. We suggest that the CD4/CD8 ratio might help to further discriminate the risk of disease progression of successfully treated HIV-infected individuals.

Mechanistically, we imagine that the maintenance of an abnormally high level of circulating CD8+ T cells could be the result of increased proliferation, decreased death, and/or changes in the rate at which these cells move between organized lymphoid structures and the peripheral blood. Although we have no evidence for increased levels of proliferation (e.g., as measured by incorporation of stable isotopes or expression of Ki67), previous studies have shown that the T_EMRA_ subset has a longer lifespan in the setting of untreated HIV disease [58], a property that may well be found in treated individuals with a low CD4/CD8 ratio. There are also data showing that progressive as well as treated HIV disease is associated with collagen deposition and loss of the fibroblastic reticular cell network within lymphoid tissue, particularly in the context of inflammation [59], [60], and such structural changes might result in the presence of an unusually large proportion of circulating CD8+ T cells. Since each of these pathologic changes is associated with a high level of inflammation, it follows that resolution of inflammation in the effectively treated individual with a low CD4/CD8 ratio might result in normalization of the ratio over time.

There are limitations to the current study that deserve mention. First, for the analysis of the correlation between the CD4/CD8 ratio in blood and GALT we used data from two clinical trials involving individuals with suboptimal CD4+ T cell recovery; hence, further studies in individuals with CD4+ T cell recovery above 500 cells/mm^3^ are needed to assess whether a low CD4/CD8 ratio reflects poor GALT immune reconstitution in these subjects. Second, although we were able to find consistent associations between CD4/CD8 ratio and T cell activation/maturation in those with high CD4+ T cell counts, we did not find such associations with plasma biomarkers (e.g., IL-6) possibly because of high assay variability; larger more definitive studies assessing this question are needed. Third, in the subset of individuals above 500 cells/mm^3^, there were only eight instances of non-AIDS related death in the Madrid cohort, and 16 in SOCA cohort, which prevented us from performing mortality analysis in this subgroup. The prognostic significance of the ratio among well-treated adults (i.e., those with undetectable viral loads and high CD4+ T cells) will need to be confirmed in larger cohorts. It will be of interest to determine in this population if the ratio has unique prognostic capacity as compared to that observed in the general population [25], [45].

Our results have potential clinical implications for novel therapeutic strategies targeting immune dysfunction in chronically treated HIV-infected individuals, in particular those with persistent expansion of CD8+ T cells despite adequate CD4+ T cell recovery. This CD4/CD8 ratio may be useful in monitoring response to therapies aimed at reducing residual immune activation, and given that prior studies have also reported that a low CD4/CD8 ratio is associated with increased markers of HIV persistence [61], [62], subjects with a high CD4/CD8 ratio may be useful targeted candidates for HIV eradication trials. Finally, ART-suppressed HIV-infected individuals who do not have an increase in the CD4/CD8 ratio might benefit from screening programs or aggressive management of concomitant risk factors for aging-associated disease.

In summary, a low CD4/CD8 ratio among ART-treated HIV-infected individuals achieving CD4+ T cell counts above 500 cells/mm^3^ may define a new clinical phenotype of individuals with higher levels of CD8+ T cell activation and senescence, depletion of naïve and enrichment for T_EMRA_ cells, increased IDO activity and higher risk morbidity and mortality. Early ART initiation may contribute to more rapid and robust CD4/CD8 ratio normalization, and the CD4/CD8 ratio may be a useful clinical endpoint to be used in evaluating novel therapies for ongoing immune dysfunction during treated infection and for HIV eradication.

## Supporting Information

Figure S1 Gating strategy for panel A and B. Data was gated using FlowJo V9. Data for both panels was first inspected for clogs or other issues that could have interrupted collection by plotting time against CD8-QDot605, this plot was also used to remove QDot-605 precipitate from analysis (not shown). Lymphocytes (i) and singlets (ii) were then gated using forward and side scatter, then live CD3+ T cells were defined on a plot of CD3 vs LIVE/DEAD Fixable Aqua Dead Cell Stain (Aqua Amine) (iii) and CD4+ and CD8+ T cells gated on a CD4 vs CD8 plot (iv). CD4+ and CD8+ T cell subpopulations were evaluated for each marker in Panel A - maturation (v) and Panel B - activation (vi) In Panel A FMO controls were used to define positive gates for expression of CCR7, CD28, CD27 and CD57. CD45RA expression was defined on a CD45 vs CD27 plot where the CD45RA gate was set high on the CD27+ cells and set according to the FMO on the CD27- cells. The boolean function in FlowJo was then used to calculate the frequency of each of the 32 possible combinations of these maturation markers on each T cell population. These Boolean populations were then used to derive the following populations for analysis: naïve (T_N_, CD45RA+CCR7+CD27+CD28+), central memory (T_CM_, CD45RA−CCR7+CD27+CD28+), transitional memory (T_TM_, CD45RA−CCR7−CD27+CD28+ and CD45RA−CCR7−CD27+CD28−), effector memory (T_EM_, CD45RA−CCR7−CD27−CD28−), and terminally differentiated (T_EMRA_, CD45RA+CCR7−CD27−CD28−). CD57 expression on each of the above populations and on total CD28− was also calculated from the boolean data. In Panel B, FMO controls were used to define positive gates for expression of CD38, HLA-DR, PD-1 and CCR5, and as for panel A, the Boolean function was used to calculate the frequency of each of the 16 possible combinations of these activation markers on each T cell population. In addition, quadrant gates were set on a CD38 vs. HLA-DR plot using FMO controls to define the frequency of CD38+HLA-DR+ cells.
(TIF)

Figure S2 Percentages and absolute counts of CD4+ T cell maturation subsets among HIV-/CMV+ individuals and ART-suppressed HIV-infected patients with CD4 counts >500 cells/mm^3^ stratified by a normal (4th quartile, ≥1) or low (1st quartile, ≤0.4) CD4/CD8 ratio. Individuals with low CD4/CD8 ratio had decreased frequencies of CD4+ T_TR_ and decreased absolute counts of T_N_, T_CM_, and T_TM_ CD4+ T cells compared to those HIV-infected patients with normal CD4/CD8 ratio and with healthy controls.
(TIF)

Figure S3 Intra-individual variability of the CD4/CD8 ratio compared to CD4+ and CD8+ T cell counts. Using data from 38 HIV-infected patients on ART-mediated HIV-RNA suppression in whom a median of 11 determinations of CD4+ and CD8+ T cells measurements were performed during a median of 81 weeks, we calculated the coefficient of variation –within subject standard deviation (blue lines) and the within subject mean (red plus symbols)– for the CD4+ T cell counts, CD8+ T cell counts and the CD4/CD8 ratio. The mean coefficient of variation was significantly lower for the CD4/CD8 ratio (12%) compared to CD4+ T cell counts (16%, P = 0.017) and for CD8+ T cell counts (18%, P = 0.001).
(TIF)

Table S1 Antibodies used for T-cell immunophenotyping.
(DOCX)

Table S2 Characteristics of chronically HIV-infected participants and HIV negative controls in SCOPE.
(DOCX)

Table S3 Characteristics of HIV-infected participants in SOCA cohort.
(DOCX)

Table S4 General characteristics of participants in the lymph node and GALT analysis.
(DOCX)

Table S5 General characteristics of OPTIONS participants.
(DOCX)

Table S6 General characteristics of participants in the Madrid cohort nested study.
(DOCX)

Table S7 Description of non-AIDS events in the Madrid cohort and causes of death in the SOCA cohort.
(DOCX)

Text S1 Additional information on the cohorts and the clinical trials analyzed in this work.
(DOCX)
