# Supplementary figures and images for: HIV-Infected Individuals with Low CD4/CD8 Ratio despite Effective Antiretroviral Therapy Exhibit Altered T Cell Subsets, Heightened CD8+ T Cell Activation, and Increased Risk of Non-AIDS Morbidity and Mortality
Source: PLoS Pathog. 2014 May 15;10(5):e1004078. doi: 10.1371/journal.ppat.1004078 (PMC4022662; doi:10.1371/journal.ppat.1004078)

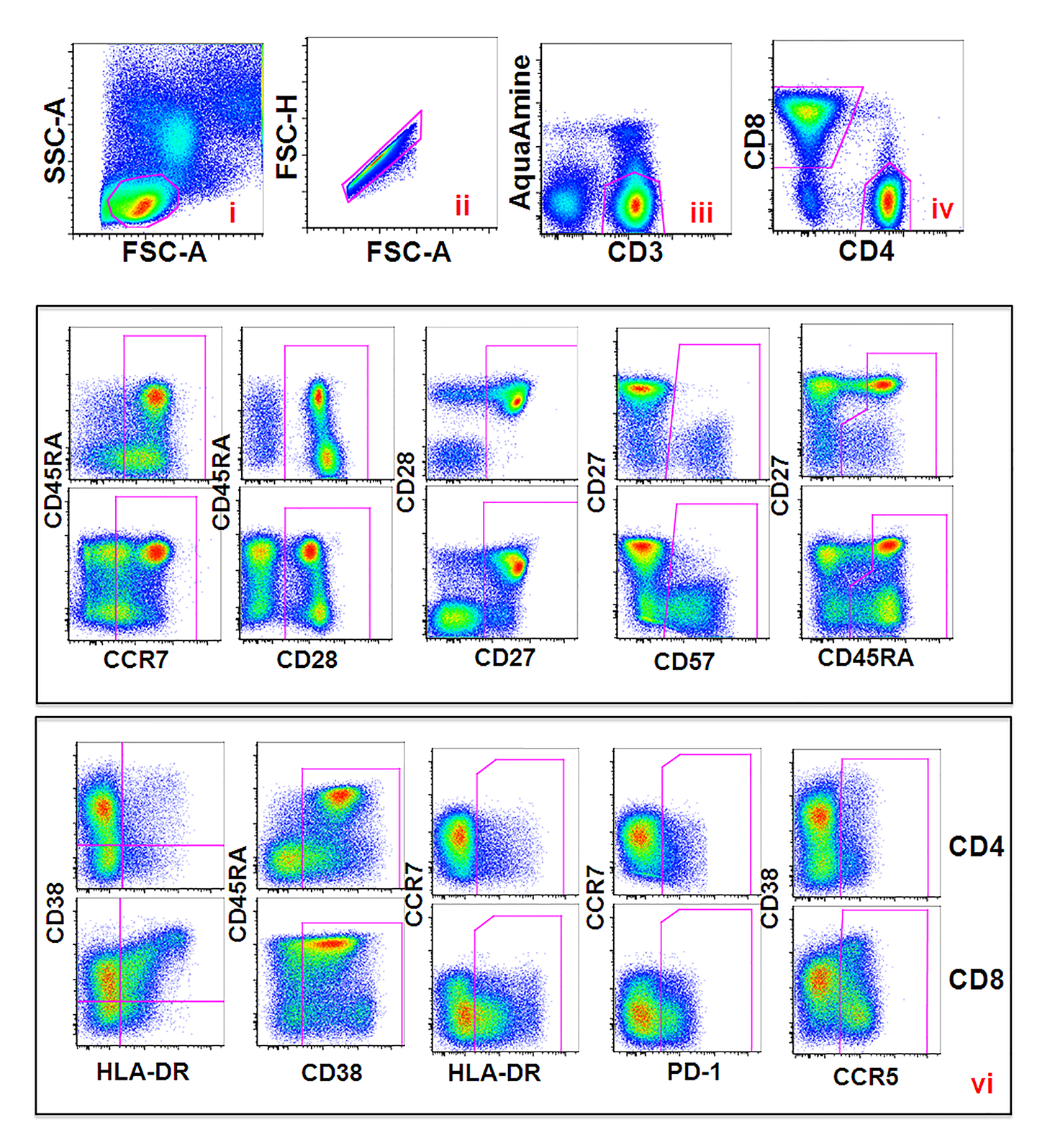

Supplement: Figure S1 — Gating strategy for panel A and B. Data was gated using FlowJo V9. Data for both panels was first inspected for clogs or other issues that could have interrupted collection by plotting time against CD8-QDot605, this plot was also used to remove QDot-605 precipitate from analysis (not shown). Lymphocytes (i) and singlets (ii) were then gated using forward and side scatter, then live CD3+ T cells were defined on a plot of CD3 vs LIVE/DEAD Fixable Aqua Dead Cell Stain (Aqua Amine) (iii) and CD4+ and CD8+ T cells gated on a CD4 vs CD8 plot (iv). CD4+ and CD8+ T cell subpopulations were evaluated for each marker in Panel A - maturation (v) and Panel B - activation (vi) In Panel A FMO controls were used to define positive gates for expression of CCR7, CD28, CD27 and CD57. CD45RA expression was defined on a CD45 vs CD27 plot where the CD45RA gate was set high on the CD27+ cells and set according to the FMO on the CD27- cells. The boolean function in FlowJo was then used to calculate the frequency of each of the 32 possible combinations of these maturation markers on each T cell population. These Boolean populations were then used to derive the following populations for analysis: naïve (TN, CD45RA+CCR7+CD27+CD28+), central memory (TCM, CD45RA−CCR7+CD27+CD28+), transitional memory (TTM, CD45RA−CCR7−CD27+CD28+ and CD45RA−CCR7−CD27+CD28−), effector memory (TEM, CD45RA−CCR7−CD27−CD28−), and terminally differentiated (TEMRA, CD45RA+CCR7−CD27−CD28−). CD57 expression on each of the above populations and on total CD28− was also calculated from the boolean data. In Panel B, FMO controls were used to define positive gates for expression of CD38, HLA-DR, PD-1 and CCR5, and as for panel A, the Boolean function was used to calculate the frequency of each of the 16 possible combinations of these activation markers on each T cell population. In addition, quadrant gates were set on a CD38 vs. HLA-DR plot using FMO controls to define the frequency of CD38+HLA-DR+ cells. (TIF) [file ppat.1004078.s001.tif]

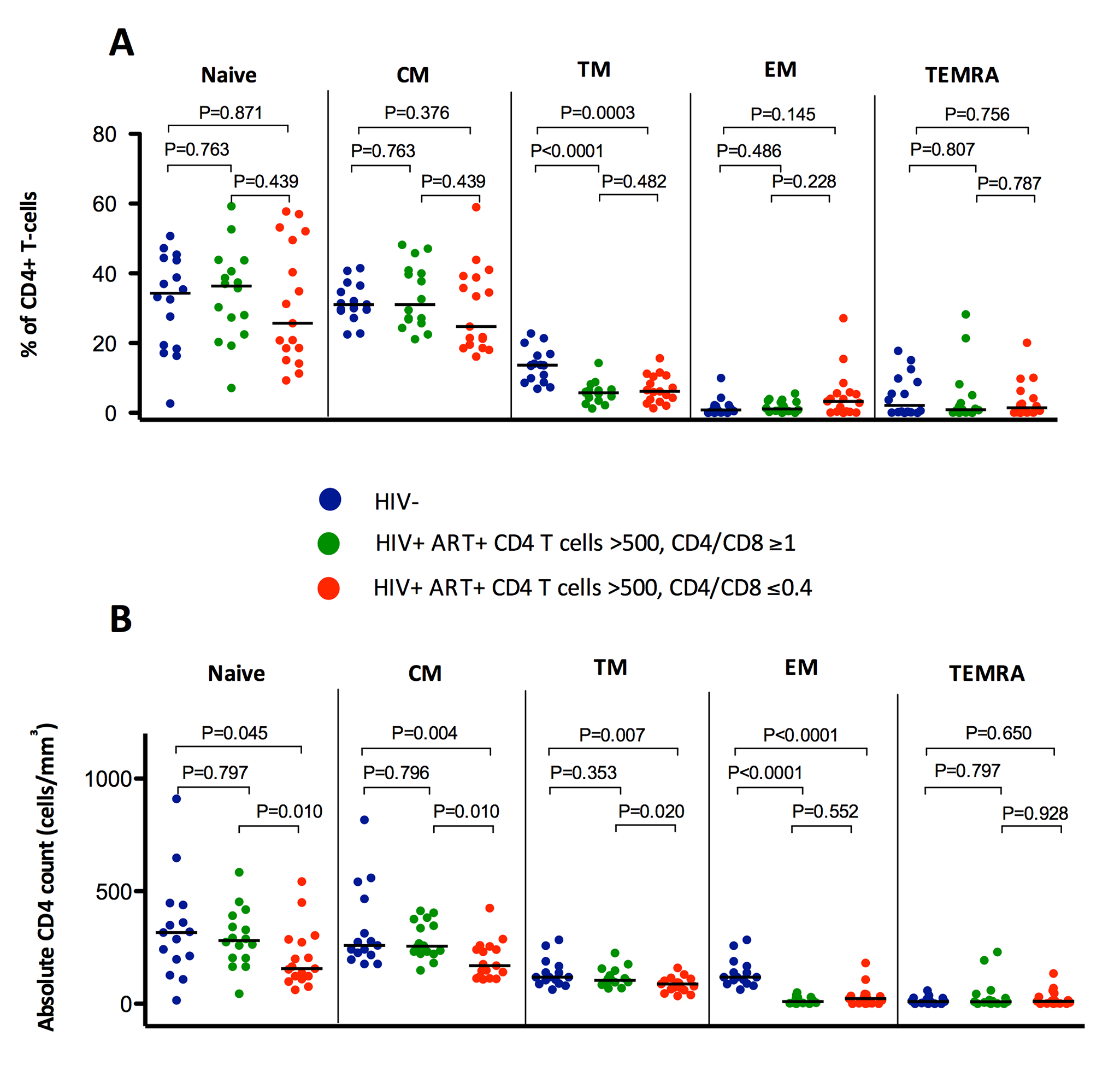

Supplement: Figure S2 — Percentages and absolute counts of CD4+ T cell maturation subsets among HIV-/CMV+ individuals and ART-suppressed HIV-infected patients with CD4 counts >500 cells/mm3 stratified by a normal (4th quartile, ≥1) or low (1st quartile, ≤0.4) CD4/CD8 ratio. Individuals with low CD4/CD8 ratio had decreased frequencies of CD4+ TTR and decreased absolute counts of TN, TCM, and TTM CD4+ T cells compared to those HIV-infected patients with normal CD4/CD8 ratio and with healthy controls. (TIF) [file ppat.1004078.s002.tif]

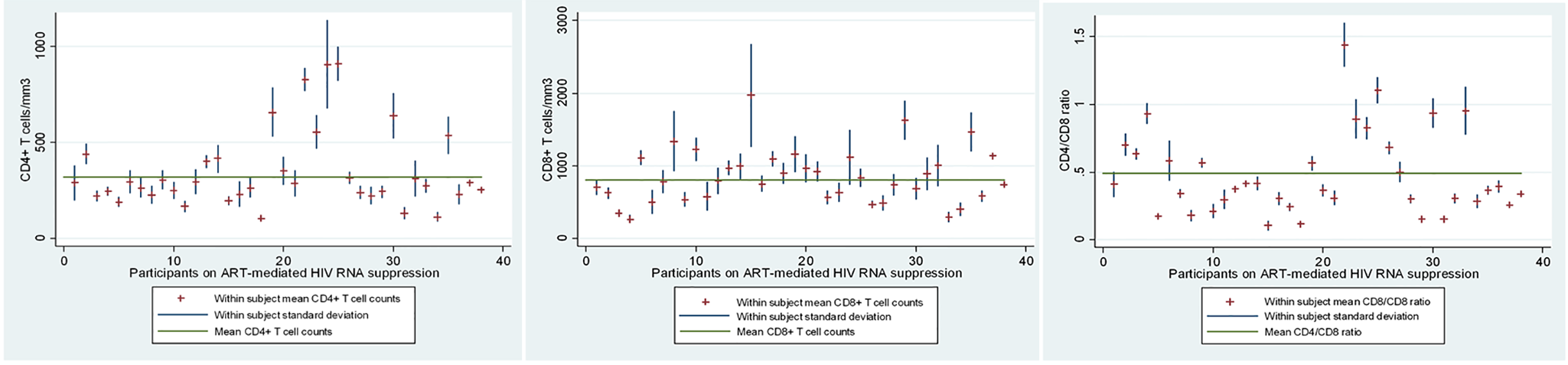

Supplement: Figure S3 — Intra-individual variability of the CD4/CD8 ratio compared to CD4+ and CD8+ T cell counts. Using data from 38 HIV-infected patients on ART-mediated HIV-RNA suppression in whom a median of 11 determinations of CD4+ and CD8+ T cells measurements were performed during a median of 81 weeks, we calculated the coefficient of variation –within subject standard deviation (blue lines) and the within subject mean (red plus symbols)– for the CD4+ T cell counts, CD8+ T cell counts and the CD4/CD8 ratio. The mean coefficient of variation was significantly lower for the CD4/CD8 ratio (12%) compared to CD4+ T cell counts (16%, P = 0.017) and for CD8+ T cell counts (18%, P = 0.001). (TIF) [file ppat.1004078.s003.tif]
