# Supplementary material for: HIV-Infected Individuals with Low CD4/CD8 Ratio despite Effective Antiretroviral Therapy Exhibit Altered T Cell Subsets, Heightened CD8+ T Cell Activation, and Increased Risk of Non-AIDS Morbidity and Mortality
Source: PLoS Pathog. 2014 May 15;10(5):e1004078. doi: 10.1371/journal.ppat.1004078 (PMC4022662; doi:10.1371/journal.ppat.1004078)
Supplement: Table S1 — Antibodies used for T-cell immunophenotyping. (DOCX) [file ppat.1004078.s004.docx]

| **Antibody/Marker** | **Conjugate** | **Source** |
| --- | --- | --- |
| **OPTIONS/SCOPE** |  |  |
| *PBMC* | | |
| **Panel A. Senescence and Maturation** | | |
| CD3 | Pacific Blue | Pharmingen |
| CD4 | PE-Texas Red | Invitrogen |
| CD8 | QDOT605 | Invitrogen |
| CD57 | Alexa Flour 647 | Biolegend |
| CD28 | PE-Cy5 | Pharmingen |
| CD45RA | ECD | BD Bioscences |
| CCR5 | PE-Cy5 | Pharmingen |
| CCR7* | PE-CY7 | Pharmingen |
|  |  |  |
| **Panel B. Activation** | | |
| CD3 | Pacific Blue | Pharmingen |
| CD4 | PE-Texas Red | Invitrogen |
| CD8 | QDOT605 | Invitrogen |
| CD38 | PE | BD Bioscences |
| HLADR | FITC | BD Bioscences |
| PD1 | Alexa 647 | BD Bioscences |
| CCR5 | PE-Cy5 | Pharmingen |
| *LNMC* |  |  |
| CD3 | V450 | Pharmingen |
| CD4 | PE-Texas Red | Invitrogen |
| CD8 | QDOT605 | Invitrogen |
| **Maraviroc trial** |  |  |
| *PBMC* |  |  |
| CD3 | Pacific Blue | Pharmingen |
| CD4 | Alexa Flour 700 | Pharmingen |
| CD8 | APC | BD Bioscences |
| MMC |  |  |
| CD3 | Pacific Blue | Pharmingen |
| CD4 | APC-Cy5.5 | Invitrogen |
| CD8 | QD605 | Invitrogen |
| **Raltegravir trial** |  |  |
| *PBMC and MMC* |  |  |
| CD3 | Pacific Blue | Pharmingen |
| CD4 | PE-Texas Red | Invitrogen |
| CD8 | Qdot 605 | Invitrogen |
| Abbreviations, PBMC, peripheral blood mononuclear cells; LNMC, lymph node mononuclear cells, MMC, mucosal mononuclear cells.  PBMC, LNMC and MMC were stained in all experiments with LIVE/DEAD Fixable Aqua Dead Cell Stain Kit (Invitrogen) to exclude non-viable cells  *In some experiments CCR7-PE-CY7 was replaced with CCR7-Alexa Flour 700 (Pharmingen) which improved CCR7 discrimination.  Data obtained with different antibodies were not combined in the same analysis. | | |

**Table S1. Antibodies used for T-cell immunophenotyping.**
