# Supplementary material for: HIV-Infected Individuals with Low CD4/CD8 Ratio despite Effective Antiretroviral Therapy Exhibit Altered T Cell Subsets, Heightened CD8+ T Cell Activation, and Increased Risk of Non-AIDS Morbidity and Mortality
Source: PLoS Pathog. 2014 May 15;10(5):e1004078. doi: 10.1371/journal.ppat.1004078 (PMC4022662; doi:10.1371/journal.ppat.1004078)
Supplement: Table S2 — Characteristics of chronically HIV-infected participants and HIV negative controls in SCOPE. (DOCX) [file ppat.1004078.s005.docx]

| **SCOPE** | **HIV-**  **(N=15)** | **HIV+ART+**  **All subjects**  **(N=95)** | **HIV+ART+**  **CD4≥500**  **(N=67)** | **HIV+ART+**  **CD4>500**  **CD4/CD8 ≤0.4**  **(N=15)** | **HIV+ART+**  **CD4≥500**  **CD4/CD8 ≥1**  **(N=16)** | **P value**  **CD4/CD8**  **≤0.4 vs.≥1** |
| --- | --- | --- | --- | --- | --- | --- |
| **Age (years, IQR)** | 43 (35-46) | 51 (45, 57) | 51 (44, 57) | 48 (44, 56) | 46 (43, 54) | 0.985 |
| **Male gender (No., %)** | 15 (94%) | 83 (87%) | 55 (83%) | 13 (87%) | 16 (80%) | 0.875 |
| **CD4+ T-cell count (cells/mm^3^, IQR)** | 863 (778, 1248) | 593 (448, 738) | 673 (578, 788) | 661 (572, 737) | 856 (672, 902) | 0.008 |
| **CD8+ T-cell count (cells/mm^3^, IQR)** | 546 (481, 665) | 1002 (778, 1420) | 1004 (813, 1468) | 1964 (1468, 2563) | 696 (594, 880) | <0.0001 |
| **CD4/CD8 ratio (IQR)** | 1.4 (1.1, 2.7) | 0.51 (0.34, 0.78) | 0.6 (0.4, 1.0) | 0.36 (0.28, 0.40) | 1.2 (1.0, 1.3) | - |
| **HIV RNA Level (log_10_copies/mL, IQR)** | NA | <1.6 | <1.6 | <1.6 | <1.6 | - |
| **Nadir CD4+ T-cell count (cells/mm^3^, IQR)** | NA | 103 (24, 493) | 52 (13, 370) | 116 (49, 851) | 35 (9, 66) | 0.005 |
| **Duration of Viral Suppression (years, IQR)** | NA | 4 (2, 8) | 5 (2, 9) | 5 (2, 7) | 7 (3, 9) | 0.386 |
| **HCV seropositivity (No., %)** | 0 (0%) | 13 (24%) | 12 (28%) | 2 (15%) | 2 (10%) | 0.772 |
| All subjects were CMV+ | | | | | | |

**Table S2. Characteristics of chronically HIV-infected participants and HIV negative controls in SCOPE.**
