# Supplementary material for: HIV-Infected Individuals with Low CD4/CD8 Ratio despite Effective Antiretroviral Therapy Exhibit Altered T Cell Subsets, Heightened CD8+ T Cell Activation, and Increased Risk of Non-AIDS Morbidity and Mortality
Source: PLoS Pathog. 2014 May 15;10(5):e1004078. doi: 10.1371/journal.ppat.1004078 (PMC4022662; doi:10.1371/journal.ppat.1004078)
Supplement: Table S3 — Characteristics of HIV-infected participants in SOCA cohort. (DOCX) [file ppat.1004078.s006.docx]

| **Table S3. Characteristics of HIV-infected participants in SOCA cohort.** | | |
| --- | --- | --- |
|  | **All Subjects**  **N=192** | **CD4+ T-cell counts**  **≥500 cells/mm^3^**  **N=49** |
| **Age (years, IQR)** | 44 (39, 51) | 44 (41, 50) |
| **Male gender (No., %)** | 154 (84%) | 39 (80%) |
| **CD4+ T-cell count (cells/mm^3^, IQR)** | 340 (213, 520) | 686 (601, 806) |
| **CD8+ T-cell count (cells/mm^3^, IQR)** | 850 (607, 1156) | 1047 (854, 1344) |
| **CD4/CD8 ratio (IQR)** | 0.4 (0.3, 0.6) | 0.7 (0.5, 0.9) |
| **HIV RNA Level (log_10_copies/mL)** | <1.6 | <1.6 |
| **Nadir CD4+ T-cell count (cells/mm^3^, IQR)** | 30 (9, 87) | 57 (8, 132) |
| **Cumulative ART exposure (years, IQR)** | 3 (1, 5) | 4 (2, 7) |
| **HCV seropositivity (No., %)** | 32 (17%) | 7 (14%) |
| **Deaths (No., %)** | 62 (34%) | 16 (33%) |
| *Abbreviations: ART, antiretroviral therapy*  *All subjects were CMV+* | | |
|  |  |  |
