# Supplementary material for: HIV-Infected Individuals with Low CD4/CD8 Ratio despite Effective Antiretroviral Therapy Exhibit Altered T Cell Subsets, Heightened CD8+ T Cell Activation, and Increased Risk of Non-AIDS Morbidity and Mortality
Source: PLoS Pathog. 2014 May 15;10(5):e1004078. doi: 10.1371/journal.ppat.1004078 (PMC4022662; doi:10.1371/journal.ppat.1004078)
Supplement: Table S4 — General characteristics of participants in the lymph node and GALT analysis. (DOCX) [file ppat.1004078.s007.docx]

**Table S4. General characteristics of participants in the lymph node and GALT analysis.**

|  | **Lymph node study**  **N=10** |  | **GALT study**  **N=32** | |
| --- | --- | --- | --- | --- |
| **Male gender (No., %)** | 10 (100%) |  | 31 (97%) | |
| **Age (years, IQR)** | 55 (51, 60) |  | 50 (45, 55) | |
| **CD4+ T-cell count (cells/mm^3^, IQR)** | 610 (467, 797) |  | 213 (170, 279) | |
| **CD8+ T-cell count (cells/mm^3^, IQR)** | 887 (678, 1382) |  | 773 (494, 1050) | |
| **CD4/CD8 ratio (IQR)** | 0.63 (0.43, 1.1) |  | 0.36 (0.24, 0.61) | |
| **HIV RNA Level, log10copies/mL** | <1.6 |  | <1.6 | |
| **Nadir CD4+ T-cell count (cells/mm^3^, IQR)** | 120 (54, 200) |  | 54 (22, 157) | |
| **Cumulative ART exposure (years, IQR)** | 13 (11, 14) |  | 4 (2, 8) | |
| Abbreviations: ART, antiretroviral therapy |  |  | |  |
