# Supplementary material for: HIV-Infected Individuals with Low CD4/CD8 Ratio despite Effective Antiretroviral Therapy Exhibit Altered T Cell Subsets, Heightened CD8+ T Cell Activation, and Increased Risk of Non-AIDS Morbidity and Mortality
Source: PLoS Pathog. 2014 May 15;10(5):e1004078. doi: 10.1371/journal.ppat.1004078 (PMC4022662; doi:10.1371/journal.ppat.1004078)
Supplement: Table S5 — General characteristics of OPTIONS participants. (DOCX) [file ppat.1004078.s008.docx]

|  | **HIV+**  **Early ART**  **N=33** | **HIV+**  **Later ART**  **N=35** |  |
| --- | --- | --- | --- |
| **Age (years, IQR)** | 38 (34, 42) | 37 (34, 43) |  |
| **Male gender, No. (%)** | 32 (97%) | 33 (0%) |  |
| **Nadir CD4 (cells/mm^3^, IQR)** | 533 (425, 735) | 314 (236, 432) |  |
| **Time on ART (years, IQR)** | 3 (2, 4) | 3 (2, 4) |  |
| **CD4+ cell count (cells/mm^3^, IQR)** | 533 (434, 742) | 567 (502, 704)*  325 (246, 435)** |  |
| **CD8+ cell count (cells/mm^3^, IQR)** | 933 (663, 1369) | 794 (615, 1134)*  874 (703, 1229)** |  |
| **CD4/CD8 ratio (IQR)** | 0.61 (0.41, 0.88) | 0.78 (0.60, 0.93)*  0.38 (0.26, 0.50)** |  |
| **ΔCD4/CD8 /y (IQR)** | +0.10 (0.07 – 0.13) | +0.03 (0.05, 0.4) |  |
| **ΔCD4 /y (cells/mm^3^, IQR)** | +51 (34, 68) | +6.6 (-13, 26) |  |
| **ΔCD8 /y (cells/mm^3^, IQR)** | -48 (-88, -9) | -29 (-75, 17) |  |
| *Abbreviations: ART, antiretroviral therapy; ΔCD4/CD8 /y, yearly variation of CD4/CD8 ratio; ΔCD4 /y, yearly variation of CD4+ T cells; ΔCD8 /y, yearly variation of CD8+ T cells*  **Recent/acute infection*  ***Pre-ART* | | | |

**Table S5. General characteristics of OPTIONS participants.**
