# Supplementary material for: HIV-Infected Individuals with Low CD4/CD8 Ratio despite Effective Antiretroviral Therapy Exhibit Altered T Cell Subsets, Heightened CD8+ T Cell Activation, and Increased Risk of Non-AIDS Morbidity and Mortality
Source: PLoS Pathog. 2014 May 15;10(5):e1004078. doi: 10.1371/journal.ppat.1004078 (PMC4022662; doi:10.1371/journal.ppat.1004078)
Supplement: Table S6 — General characteristics of participants in the Madrid cohort nested study. (DOCX) [file ppat.1004078.s009.docx]

**Table S6. General characteristics of participants in the Madrid cohort nested study.**

|  | **Cases**  **N=33** | **Controls**  **N=33** | **P value** |
| --- | --- | --- | --- |
| **Male gender (No., %)** | 27 (82%) | 27 (82%) | - |
| **Age (years, IQR)** | 46 (41, 49) | 43 (41, 47) | 0.682 |
| **CD4+ Count (cells/mm^3^, IQR)** | 666 (580. 875) | 714 (583, 837) | 0.841 |
| **CD8+ Count (cells/mm^3^, IQR)** | 1239 (974, 1494) | 897 (687, 1108) | 0.002 |
| **CD4/CD8 ratio (IQR)** | 0.55 (0.44, 0.75) | 0.81 (0.67, 0.94) | 0.002 |
| **HIV RNA Level, log_10_copies/mL** | <1.6 | <1.6 | - |
| **Nadir CD4+ Count (cells/mm^3^, IQR)** | 221 (80, 303) | 213 (114, 289) | 0.680 |
| **Cumulative ART exposure (cells/mm^3^, IQR)** | 10 (6, 12) | 6 (5, 7) | 0.001 |
| **HCV seropositivity (No, %)** | 7 (21%) | 5 (15%) | 0.523 |
| Abbreviations: ART, antiretroviral therapy | | | |
