# Supplementary material for: HIV-Infected Individuals with Low CD4/CD8 Ratio despite Effective Antiretroviral Therapy Exhibit Altered T Cell Subsets, Heightened CD8+ T Cell Activation, and Increased Risk of Non-AIDS Morbidity and Mortality
Source: PLoS Pathog. 2014 May 15;10(5):e1004078. doi: 10.1371/journal.ppat.1004078 (PMC4022662; doi:10.1371/journal.ppat.1004078)
Supplement: Table S7 — Description of non-AIDS events in the Madrid cohort and causes of death in the SOCA cohort. (DOCX) [file ppat.1004078.s010.docx]

**Table S7. Description of non-AIDS events in the Madrid cohort and causes of death in SOCA cohort.**

|  | **Madrid cohort**  **Non-AIDS event (N=33)**  **(No., %)** | **SOCA cohort**  **Cause of death**  **(N=62)**  **(No., %)** |
| --- | --- | --- |
| **Cardiovascular** | 17 (52%) | 17 (27%) |
| **Non-AIDS cancer** | 11 (33%) | 8 (13%) |
| **End-stage liver disease** | 4 (12%) | 5 (8%) |
| **End-stage renal disease** | 1 (3%) | 3 (5%) |
| **Respiratory arrest** | NA | 2 (3%) |
| **AIDS-related** | - | 5 (8%) |
| **Infection** | - | 6 (10%) |
| **Unknown** | - | 16 (26%) |

Only eight instances of non-AIDS related death in the Madrid cohort (median CD4+ T cell count 666 cells/mm^3^), and 16 in SOCA cohort (median CD4+ T cell count 340 cells/mm^3^)
