# Supplementary material for: HIV-Infected Individuals with Low CD4/CD8 Ratio despite Effective Antiretroviral Therapy Exhibit Altered T Cell Subsets, Heightened CD8+ T Cell Activation, and Increased Risk of Non-AIDS Morbidity and Mortality
Source: PLoS Pathog. 2014 May 15;10(5):e1004078. doi: 10.1371/journal.ppat.1004078 (PMC4022662; doi:10.1371/journal.ppat.1004078)
Supplement: Text S1 — Additional information on the cohorts and the clinical trials analyzed in this work. (DOCX) [file ppat.1004078.s011.docx]

**TEXT S1. METHODS**

**Subjects**

Study participants were sampled from four cohorts and two clinical trials:

1) SCOPE: a clinic-based cohort of over 1500 chronically HIV-infected participants and HIV-uninfected controls in University of California San Francisco, aimed at investigating the long-term clinical and immunological consequences of HIV infections and their treatment. We used data from subjects with available immunophenotyping in PBMC. We also used data from a sub-study aiming to determine markers of viral persistence from long-term ART treated patients in lymph node biopsies.

2) The Study of the Ocular Complications of AIDS (SOCA): a multicenter cohort of over 2200 HIV-infected participants who initiated ART with an AIDS diagnosis. In the nested case-control analysis of immunologic predictors of mortality in the SOCA cohort, cases (N=62) who died of non-accidental death and had PBMC and plasma samples available within 18 months of death with confirmed plasma HIV RNA levels <400 copies/ml were each matched to two controls (N=130) by age, gender, duration of viral suppression, history of CMV retinitis, and nadir CD4+ cell count.

3) OPTIONS: a prospective cohort formed in 1996, enrolling patients <12 months after HIV antibody seroconversion (since 2003, this was restricted to <6 months post-seroconversion) [1]. Early HIV is diagnosed if participants meet any of the following criteria: (A) two plasma HIV-1 RNA levels ≥3000 copies/mL with a negative or indeterminate HIV-1 antibody test; (B) positive HIV-1 antibody test with history of negative HIV-1 antibody test ≤12 months prior (in 2003, this was changed to ≤6 months); or (C) clinical history suggesting recent HIV acquisition along with reactive standard HIV-1 antibody test, but nonreactive less sensitive (“de-tuned”) HIV-1 antibody test. Standardized estimated infection dates are calculated using these data. As previously described [1], OPTIONS participants who fitted in the following categories were selected: (A) Early ART group: participants who initiated ART <6 months after estimated HIV infection date and had undetectable plasma RNA values for ≥2 years (continuous ART-mediated virologic suppression); (B) Later ART group: participants who initiated ART at least 2 years after estimated HIV infection date and had ≥2 years of virologic suppression on ART.

4) The Madrid cohort: a clinic-based cohort formed in 1999 of over 2400 HIV-infected patients who initiated ART in University Hospital Ramón y Cajal, Madrid. We sampled HIV-infected adults during at least one year who achieved HIV RNA <50 copies/mL. Cases (N=130) were subjects who had experienced serious non-AIDS events, as defined as ischemic heart disease (myocardial infarction, percutaneous coronary angioplasty, coronary artery bypass surgery), stroke (ischemic or hemorrhagic) or non-AIDS defining malignancies (excluding non-melanomatous skin cancers), end-stage liver disease and end-stage renal disease. Controls (n=400) were ART-suppressed unmatched patients at least one year under HIV suppression. Censoring occurred at the time of event in cases and at the last clinic visit in controls until September 2012.

5) The raltegravir (NCT00631449) intensification in ART-treated HIV-infected patients randomized placebo-controlled clinical trial [2]: Thirty treated subjects with CD4+ T cell counts of <350 cells/mm^3^despite viral suppression for >1 year were randomized to add raltegravir (400 mg twice daily) or matching placebo for 24 weeks. The primary end points were the proportion of subjects with undetectable plasma viremia (determined using an ultrasensitive assay with a lower limit of detection of 3 copy/mL) and a change in the percentage of CD38+HLADR+CD8+ T-cells in PBMC. Colorectal biopsies were performed in a subset of participants, who where included in this study.

6) The maraviroc (NCT00735072) intensification in ART-treated HIV-infected patients randomized placebo-controlled clinical trial [3]: 45 HIV-infected subjects with CD4 counts <350 cells/mm^3^ and plasma HIV RNA levels <48 copies per mL on ART were randomized to add maraviroc or matching placebo to their regimen for 24 weeks followed by 12 weeks on ART alone. The primary analysis compared the week 24 changes from baseline in the percentage of CD38+HLADR+CD8+ T-cells between maraviroc and placebo-treated subjects. Colorectal biopsies were-performed in a subset of participants, who where included in this study.

**References**

1. Jain V, Hartogensis W, Bacchetti P, Hunt PW, Hatano H, et al. (2013) Antiretroviral Therapy Initiated Within 6 Months of HIV Infection Is Associated With Lower T-Cell Activation and Smaller HIV Reservoir Size. J Infect Dis 208: 1202–1211. doi:10.1093/infdis/jit311.

2. Hatano H, Hayes TL, Dahl V, Sinclair E, Lee T-H, et al. (2011) A randomized, controlled trial of raltegravir intensification in antiretroviral-treated, HIV-infected patients with a suboptimal CD4+ T cell response. J Infect Dis 203: 960–968. doi:10.1093/infdis/jiq138.

3. Hunt PW, Shulman NS, Hayes TL, Dahl V, Somsouk M, et al. (2013) The immunologic effects of maraviroc intensification in treated HIV-infected individuals with incomplete CD4+ T-cell recovery: a randomized trial. Blood 121: 4635–4646. doi:10.1182/blood-2012-06-436345.
